# Nanofluorapatite Hydrogels in the Treatment of Dentin Hypersensitivity: A Study of Physiochemical Properties and Fluoride Release

**DOI:** 10.3390/gels9040271

**Published:** 2023-03-25

**Authors:** Katarzyna Wiglusz, Maciej Dobrzynski, Martina Gutbier, Rafal J. Wiglusz

**Affiliations:** 1 Department of Basic Chemical Sciences, Faculty of Pharmacy, Wroclaw Medical University, Borowska 211 A, 50566 Wroclaw, Poland; 2 Department of Pediatric Dentistry and Preclinical Dentistry, Faculty of Dentistry, Wroclaw Medical University, Krakowska 26, 50425 Wroclaw, Poland; 3 Institute of Low Temperature and Structure Research, Polish Academy of Sciences, Okolna 2, 50422 Wroclaw, Poland

**Keywords:** nanofluorapatite hydrogels, fluorides, dental hypersensitivity, nanosized fluorapatite, fluoride release profile

## Abstract

The aim of this work was to prepare a new hydrogel based on nanohydroxyapatite (nFAP, 10% *w/w*) and fluorides (4% *w/w*), both of which are used as sources of fluoride ions in the treatment of dentin hypersensitivity, and to characterize its physicochemical properties. The release of fluoride ions from 3 gels (G-F, G-F-nFAP, and G-nFAP gel) was controlled in Fusayama–Meyer artificial saliva at pH 4.5, 6.6, and 8.0. The properties of the formulations were determined by an analysis of viscosity, a shear rate test, a swelling study, and gel aging. Various methods, i.e., FT-IR spectroscopy, UV-VIS spectroscopy, and thermogravimetric, electrochemical, and rheological analysis, were used for the experiment. The profiles of fluoride release indicate that the amount of fluoride ions released increases with a decrease in the pH value. The low pH value facilitated water absorption by the hydrogel, which was also confirmed by the swelling test, and it promoted the exchange of ions with the surrounding environment. Under conditions similar to physiological conditions (at pH 6.6), the amounts of fluorides released into artificial saliva were approximately 250 µg/cm^2^ and 300 µg/cm^2^ for the G-F-nFAP hydrogel and G-F hydrogel, respectively. The aging study and properties of the gels showed a loosening of the gel network structure. The Casson rheological model was used to assess the rheological properties of the non-Newtonian fluids. Hydrogels consisting of nanohydroxyapatite and sodium fluoride are promising biomaterials in the prevention and management of the dentin hypersensitivity.

## 1. Introduction

A frequent complaint presented by patients visiting the dental office is pain and discomfort related to tooth wear and dentine hypersensitivity [1,2]. Despite significant progress being made in regards to dental materials and treatment techniques, these two pathological terms are prevalent in the world of dentistry and are closely associated with each other. This can be explained by the fact that dentinal hypersensitivity only occurs in situations where the inner sensitive layer of teeth, i.e., dentine, is exposed to the environment of the oral cavity due to the loss of the enamel, the outer protective coating of the tooth. Additionally, the dentine of individuals suffering from hypersensitivity is characterized by dentinal tubules that are higher in number and larger in diameter compared to the non-sensitive dentine of non-affected patients [3]; in these patients, the stimulation of the nerve cells does not occur, thus making dentine insensitive to stimuli such as tooth brushing, airflow or cold.

Tooth wear may have a chemical or mechanical etiological background and can commonly be classified as erosion, abrasion, or attrition. A diet rich in sweets and sour fruit, excessive alcohol consumption, pregnancy sickness, or bulimia are all factors contributing to chemical tooth wear, i.e., erosion [4]. Mechanical damage from interactions with objects other than tooth-to-tooth contact, such as tooth brushing, dental treatments (scaling and root planning), or nail biting, causes abrasion. Excessive clenching and grinding of the teeth may lead to dental attrition [5,6,7]. Clinical observations show that the aforementioned mechanisms rarely act alone and commonly interact with each other [8].

Once dentinal tubules are exposed, the development of possible dentin hypersensitivity can mainly be explained by two theories. The first hypersensitivity hypothesis is based on the stimulation of the odontoblasts, which are located between the dentin and the pulp with their endings penetrating the dentinal tubules. Pain occurs after the odontoblasts come in contact with the pulp nerve endings [9,10]. The second, more widely accepted theory is related to the hydrodynamic mechanism; this theory states that the pain is caused by the flow of fluid in the dentinal tubules, which is induced by external factors [11,12].

The management of dentin hypersensitivity focuses on prevention and proper oral hygiene habits, and treatment by the obliteration of dental canaliculi. In dental practice, there are many widely used oral and dental formulations [13,14], e.g., pastes, mouth rinses, gels, dental adhesives, and varnishes. Dental preparations contain fluoride ions and oxalate ions, which then precipitate on the surface of the teeth or in the tooth tubules in the form of insoluble compounds, i.e., calcium fluoride, calcium oxalate [15], strontium chloride and acetate [16], potassium nitrate [17], hydroxyapatite [18,19] and fluorapatite [20]. The mechanism of these preparations is related to the blockage of the dental tubules by the crystallized components [21] or by covering the tooth with a special isolating film, penetrating several dozen micrometers into the exposed dentinal tubules [22]. Moreover, potassium nitrate probably inhibits the sensation of pain by disrupting stimulus conduction by the depolarization of the membrane of the nerve fibers [17].

Fluorapatite, with the formula Ca_10_(PO_4_)_6_F_2_, is a biocompatible material [23] and therefore has the potential to be studied as a material for use in dental practice; it also lends itself to research into reducing dentin hypersensitivity. Earlier experiments with fluorapatite indicate its remineralizing function [24], which supports the osseointegration of implants [25], increases the mechanical parameters of glass ionomer cements for tooth reconstruction [26], and suppresses pain perception [27]. Hydrogel formulations containing a combination of fluorapatite and sodium fluoride, containing a high level of 20,000 ppm fluoride ion, have not yet been analyzed. Therefore, our studies focused on a new hydrogel consisting of fluorapatite and sodium fluoride, which are both known to have an effect in reducing hypersensitivity [28]. Fluorapatite and sodium fluoride are fluoride ion donors that are found in the gel base prepared from a water-soluble and pH-sensitive polymer Carbomer 974, which is a bioadhesive [29,30,31], synthetic polymer of acrylic acid with the formula (CH_2_-CHCO_2_H)_n_ [32]. The use of a bioadhesive material is appropriate for the controlled release of fluoride ions, which, as the literature proves, have an occlusive effect on dental tubules, thereby protecting the pulp from external stimuli. The Carbopol polymers demonstrate low toxicity [33]; thus, the polymer is able to release bioactive compounds dispersed in the polymer matrix.

Other studies have shown that effective treatment with long-term results has been related to the intratubular deposition of mineral crystals [32]. The superficial closure of dentinal tubules with a fluoride-calcium precipitate only leads to temporary blocking of the openings, providing short-term relief from hypersensitivity as the precipitate can either be removed by daily tooth brushing or dissolved by the consumption of acidic beverages.

The aim of this work was to prepare and characterize the properties of a new hydrogel based on nanohydroxyapatite and fluorides. The nanosized fluorapatite crystals contained in the hydrogel are of sufficient small size to potentially occlude the dentinal tubules in more depth. A study comparing the effectiveness of desensitizing agents in the occlusion of dentinal tubules found that an experimental hydroxyapatite-based composition was the longest lasting. Its mineral layer formed at the dentine interface seemed to be highly durable; both larger and smaller crystals were still present after rinsing for 24 h, 48 h, and 7 days in the artificial saliva solution, according to SEM analysis [34]. The G-F-nFAP hydrogel is to be expected to have a similar effect and longevity, but this still needs to be proven in in vitro experiments on human teeth.

The experiments were performed to determine the physicochemical properties of the gels, as well as the nature of their fluoride ion release. Analysis of viscosity, a shear rate test, a swelling study, and gel aging were carried out using various methods, i.e., FT-IR spectroscopy, UV-VIS spectroscopy, and thermogravimetric, electrochemical, and rheological analysis.

## 2. Results and Discussion

### 2.1. Physicochemical Analysis of the Fluorapatite Compound

**Figure 1:**
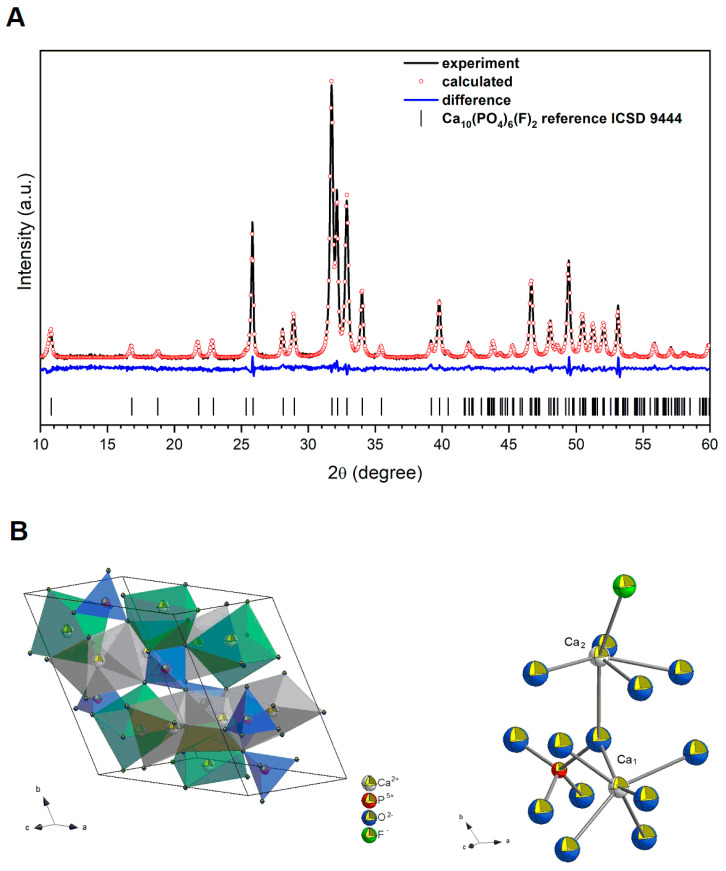
XRD pattern (back line) and result of the Rietveld analysis of the Ca_10_(PO_4_)_6_(F)_2_ nanoparticles annealed at 450 °C (**A**); projection of the obtained Ca_10_(PO_4_)_6_F_2_ unit cell and indications of the Ca_1_ and Ca_2_ crystallographic positions (**B**).

The characterization of the Ca_10_(PO_4_)_6_F_2_ sample was carried out by means of powder XRPD measurement (see Figure 1). We can observe that a pure hexagonal phase (with space group P63/m), corresponding to the standard reference of the fluorapatite (ICSD-9444) [35], was obtained and underwent heat treatment at 450 °C.

**Figure 2:**
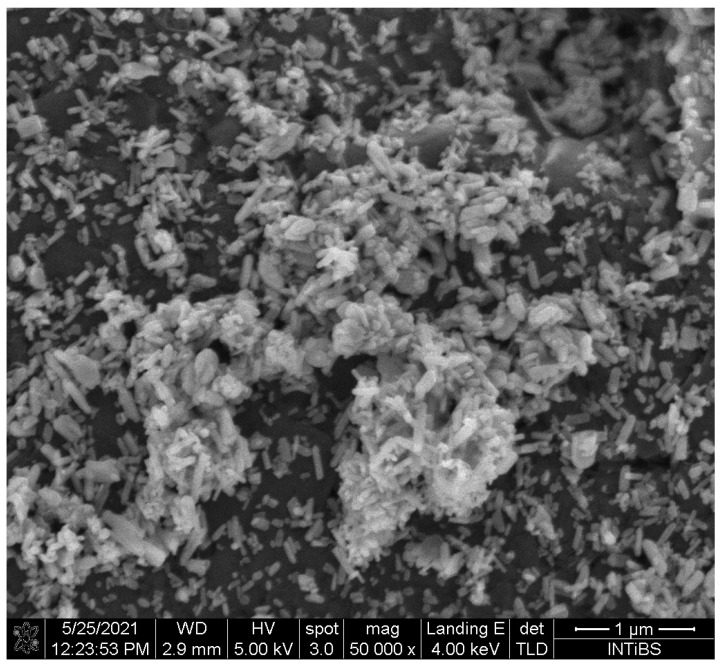
Scanning electron microscope (SEM) image of nanosized fluorapatite.

Moreover, the unit cell parameters for the obtained sample were calculated using the Rietveld method [36] with the anisotropic approach using Maud 2.99 software [37]. The difference in the intensity scale in the line (Y_Obs_–Y_Calc_) is close to zero, indicating a good correlation between the observed and theoretical XRPD patterns (Figure 1). No additional secondary phases, impurities, or amorphous forms were detected, confirming the formation of the designated phase. The quality of structural refinement was also evaluated according to R values, with the results shown in Table 1. The crystallite size of the studied material was estimated to be 57.85 nm (Figure 2).

The parameters were optimized as follows: scale factor, background with exponential shift, exponential thermal shift and polynomial coefficients, basic phase, microstructure, crystal structure, size strain (anisotropic, no rules), structure solution model (genetic algorithm SDPD), shift lattice constants, profile half-width parameters (u, v, w), texture, and lattice parameters (a, b, c), as well as factor occupancies and atomic site occupancies (Wyckoff).

Additionally, the projection of the obtained nanosized fluorapatite unit cell and the coordination polyhedra of calcium ions are presented in Figure 1B. There are two non-equivalent Ca^2+^ sites, of which Ca_1_ is coordinated by nine oxygen atoms derived from phosphate groups and Ca^2+^ ion is coordinated by six oxygen atoms derived from phosphate groups, as well as one fluoride ion.

### 2.2. The Physicochemical Characteristics of the Gels

#### 2.2.1. The Rheological Test

The rheological behavior is important for characterizing the properties of the gels. It affects the method of application and the gel layer thickness. The three tested gels have the same content of suspended Carbopol, but they differ with regards to the additional component (i.e., the source of fluoride), and therefore presented different mechanical properties. It is the presence of fluorapatite that seems to be the differentiating factor. The rheograms of the gels indicated non-Newtonian fluids (change in viscosity dependent upon the degree of shear applied), presenting pseudoplastic characteristics and shear thinning.

Shear stress causes the destruction of intermolecular forces (van der Waals forces, hydrogen bonds, dipole–dipole type), which is demonstrated by a reduction in viscosity. The data were analyzed using the Casson rheological model [38] Equation (1), with a regression coefficient higher than 0.9980:

(1) $$\tau^{1/2}=\tau_{0}^{1/2}+\eta^{1/2}\cdot {\dot{\gamma }}^{1/2}$$

where *τ* is the shear stress, *τ*_0_ is the yield stress, *η* is the viscosity, and $\dot{\gamma }$ is the shear rate.

In hydrogels, intermolecular forces between particles (van der Waals forces, dipole–dipole interactions, and hydrogen bonds) create a three-dimensional structure [39,40]. Under the influence of shear stress, the yield point is exceeded and the intermolecular forces are broken down. The gels start to flow (the material behaves like a fluid), which is demonstrated by a reduction in viscosity.

Figure S1 (in the Supplementary Materials) shows the function shear stress vs. the shear rate of the hydrogels. The rheograms show different values of yield points, which are dependent on the composition of the gels. The G-nFAP gel obtained the highest yield point value (182.34 ± 12.20 Pa, at 25 °C, Table 2).

The slightly lower value of 178.01 ± 9.13 Pa for the G-F-nFAP gel compared to the G-nFAP gel is associated with the presence of nFAP, which stabilizes the hydrogel. Meanwhile, in the case of the G-F gel, the viscosity and the yield points were reduced.

**Figure 3:**
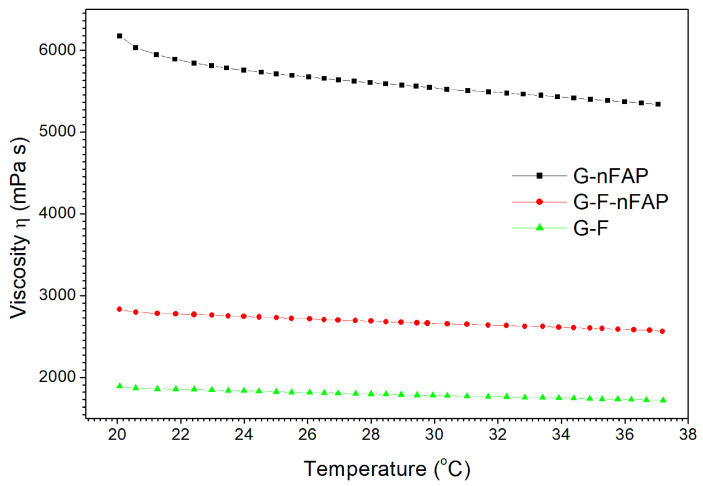
Dependence of the viscosity on the temperature of the examined gels at 150 s^−1^.

The temperature curve of the G-nFAP gel differs in comparison to the G-F-nFAP and G-F gels (Figure 3). The highest viscosity value is characteristic of the G-nFAP gel and amounts to 5340 mPa·s (at 37 °C). The dynamic viscosities were lower in the case of the G-F-nFAP gel and the G-F gel, and amount to 2562 mPa·s and 1720 mPa·s, respectively.

The viscosity of the gels mainly depends on their compounds, which influence intermolecular interactions and the gels’ networks. On the other hand, the influence of temperature is insignificant, ranging from about 13.5 to 9.0% with a temperature increase of 17 degrees (from 20 to 37.5 °C), for the G-nFAP, G-F-nFAP, and G-F gels.

The viscosity can slow down the rate of sedimentation of the components of the hydrogels, whereas a high yield value is necessary to create permanent suspensions. Viscosity and yield stress do not vary significantly with temperature changes.

#### 2.2.2. The Swelling Study

**Figure 4:**
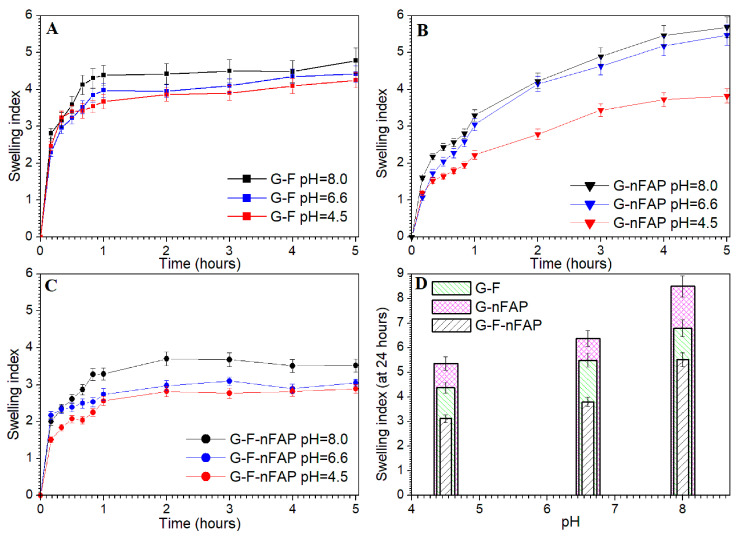
Swelling tests of the hydrogels in Fusayama–Meyer artificial saliva buffer (pH 4.5, 6.6, and 8.0) at 37 °C for G-F gel (**A**), G-nFAP gel (**B**) and G-F-nFAP gel (**C**) and a comparison of the swelling of the gels during the 24 h experiment (**D**) at different pH values.

The swelling behavior of the hydrogels was tested for 6 h in near-physiological conditions at pH values ranging from 4.5 to 8.0 and at a temperature of 37 °C. The hydrogels, i.e., G-F, G-F-nFAP, and G-nFAP, were examined in their dried form. The gels absorbed the buffer quickly in the first hour of the test. The G-F gel showed a particularly intensive absorption of the buffer solution (Figure 4A). The behaviors of the G-nFAP gel and the G-F-nFAP gel were different (Figure 4B,C). During the 6 h test, the swelling indexes increased to the values of 3.5–4.5 and 3.5–5.5, respectively.

The swelling curves of the G-F-nFAP gels were stable after 2 h of the experiment. On the other hand, the curves of the G-nFAP gels rose the whole time. A significant correlation between the pH and the value of the swelling index was observed. As the pH value increased, the swelling of the gels decreased. This is because the degree of cross-linking of the gels determines the swelling capacity.

The alkaline environment of the buffer promotes the gelation of the samples. The swelling tendency of the gels continued after the 24 h of the experiment (Figure 4D). The highly cross-linked systems have a compact structure; then, the polymer chains reduce mobility and the degree of swelling is reduced [41]. In an acidic environment (pH 4.5), the stability of the gels is lowered due to the protoning of carboxyl groups and the reduction in the negative charge.

#### 2.2.3. Thermogravimetric Analysis

The thermogravimetric stability of the gel formulation is shown in Figure S2 (in the Supplementary Materials). The first thermal loss was from 25 to 180 °C, with a mass loss of 75–85% assigned to the adsorbed water and H-bounded water. The second thermal loss reached a maximum at 430 °C and corresponded to the destruction of the interaction between the gel’s components, as well as the thermal decomposition of the backbone chains of the Carbopol polymer and the release of volatile products. 

#### 2.2.4. The Control of the Gels’ Network

**Figure 5:**
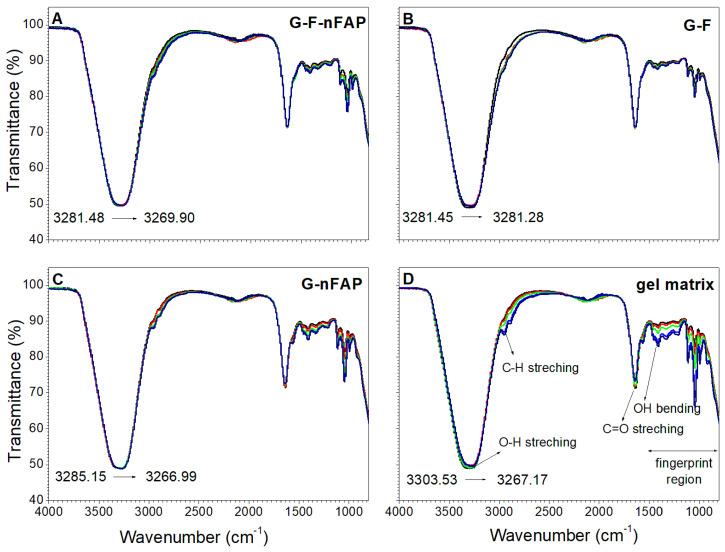
The FT-IR spectra of the gel formulations (G-F-nFAP gel (**A**), G-F gel (**B**), G-nFAP gel (**C**), and gel matrix (**D**). The gels were tested during the first (black), second (red), third (green), fourth (blue), and fifth (navy line) weeks of the experiment.

Changes in the structure of the hydrogel matrix were tracked using infrared spectroscopy, which showed changes within the oscillatory vibrations of the OH group. The Carbopol polymer forms hydrogen bonds with the gel components: fluorapatite and sodium fluoride. The spectra of the Carbopol formulations (Figure 5) show that the characteristic carbonyl stretching vibrations (C=O) peak at 1640 cm^−1^ and the C–H stretching vibrations appear between 3200 and 2800 cm^−1^. The characteristic carboxylic acid O–H single bond stretching overlaps with the C–H region to some degree. The hydrogen-bonded O–H indicates the presence of the OH groups associated with the stretching vibrations of hydrogen bonds (3300–2500 cm^−1^) and OH bending at about 1440–1395 cm^−1^ (Figure 5D).

The position of the stretching vibrations of the OH groups changed for the hydrogels throughout the tests carried out during the five weeks of the experiment. The band redshifted to lower frequencies from 3281 to 3269 cm^−1^ in the case of G-F-nFAP (Figure 5A) and 3285 to 3267 cm^−1^ for G-nFAP (Figure 5C), which is taken as evidence of hydrogen bonding and an increase in the O–H---O distance, indicating a weakening of this bond. Additionally, the fluoride ion interacts with the structure of the gel to form strong hydrogen bonds. For the G-F formulation (Figure 5B), the changes were negligible and the stretching vibration of the OH group was observed at 3281 cm^−1^. Carbopol is a well-studied polymer that is known to degrade when exposed to light and certain chemical agents [42]. The thermal degradation products of Carbopol were described by Kanis et al. [43]. As a result of the fragmentation of the structure and the decarboxylation of the Carbopol acid groups, carbon dioxide is produced. In our study, unsaturated structures (associated with a band at 1650 cm^−1^) were observed in the fifth week of the experiment.

In summary, the five-week aging study of the gels showed a loosening of the gels’ structure. The study indicates that the non-associated hydrogen bonds play a more significant role as compared to the inter- and intramolecular bonds. This proves the loosening of the gel structure and facilitates the release of the bound molecules.

### 2.3. Release Experiments

The subjects of this experiment were three hydrogels with the potential to remineralize enamel [44] and reduce the symptoms of dentin hypersensitivity. The G-F gel contained 4% *w/w* NaF (with F^-^ 20,000 ppm), the G-F-nFAP gel had a combination of 4% *w/w* NaF and 10% *w/w* nFAP, and the G-nFAP gel only had 10% *w/w* nFAP. The study was based on an assessment of the release of fluoride ions from the gel matrix into the acceptor fluid (the artificial saliva) at different pH values. Three different levels of pH were used to resemble the environment of the oral cavity. The following values were chosen: 4.5 for a low pH, which simulated acidic saliva in the presence of acids from the diet, a physiological pH of 6.6 (normal pH ranges from 6.2–7.6 [45]), and a pH of 8.0 (pH that promotes the growth of microorganisms, causing periodontal disease [45]). The amounts of fluoride released were significantly higher in the studied solutions with a pH of 4.5. The G-F gel with 4% *w*/*w* of sodium fluoride showed a higher release rate in comparison to the other hydrogels and reached a value of about 70% after 5 weeks.

**Figure 6:**
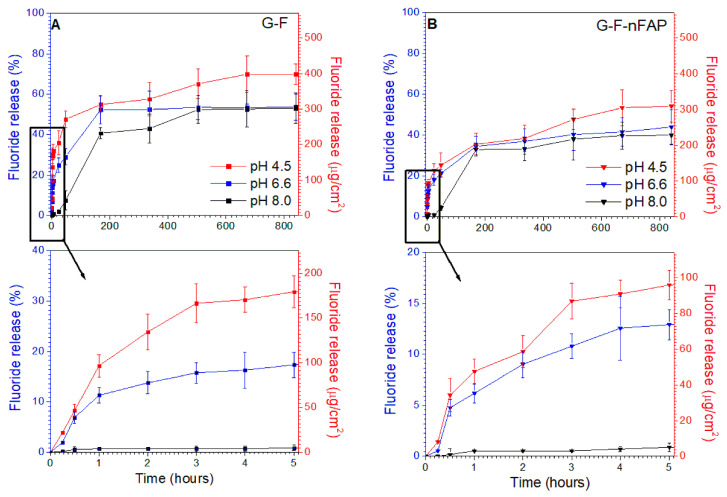
The fluoride release test in the artificial saliva for gel G-F (**A**) and gel G-F -nFAP (**B**) at different pH values (4.5, 6.6, and 8.0) and a temperature of 37 °C.

Figure 6A shows the release profile of the G-F gel at different pH values from a long-term perspective (five weeks), as well as during the first six hours of the release experiment. It can be noted that a significant amount of fluoride was released during the first week of the study (up to 168 h), after which the levels of fluoride released reached 55, 53, and 41% at pH 4.5, 6.6, and 8.0, respectively. After 3 weeks of the experiment (504 h), the concentrations of F^-^ reached a steady state with time; however, particularly at a low pH, the fluoride release levels were still rising after 4 weeks. After 5 weeks of the experiment, the measured levels were 70, 54, and 53% with increasing pH.

Regardless of the experimental conditions, it was noted that the addition of nFAP slowed down the release of fluoride (Figure 6B). After 5 weeks, the levels of fluoride released from the G-F-nFAP gels containing a mixture of NaF and nFAP ranged from 54 and 44 to 40%, at pH values of 4.5, 6.6, and 8.0, respectively.

It is certain that the presence of fluorapatite changes the physical properties of the gel by increasing its viscosity compared to the G-F gels without nFA; this may be relevant to the release process.

According to this research, the change in viscosity had an important influence on the release process. Additionally, Figure 6 shows that a significant amount of fluoride is released during the first week of the study (up to 168 h). Subsequently, the amount of fluoride is at a level of about 30% in the case of nFAP-doped gels (G-F-nFAP), whereas the G-F gel showed levels of 55, 53, and 41% at a pH 4.5, 6.6, and 8.0, respectively. After 3 weeks of the experiment (504 h), the concentrations of F^-^ reached a steady state.

This study of new hydrogels containing nanohydroxyapatite and sodium fluoride focused on the potential reduction in dentine hypersensitivity. The application of conditions imitating the environment of the oral cavity, with the Fusayama–Meyer artificial saliva solution, showed the behavior of the gels. Due to the warm intraoral environment (the normal average oral temperature being 37 °C [46]), the gel has a relatively low viscosity compared to its initial application form (stored at room temperature) and can be dosed easily (Figure 3). It may be helpful to apply and spread the gel over the whole surface of the tooth, including hard-to-reach places.

The study of the swelling test (Figure 4) and the increase in the gelation process of the Carbopol matrix with the increase in the viscosity of the system reduce the release of fluoride ions. On the other hand, the destabilization of the gel structure (Figure 5) caused by the lower pH value of the saliva, and thus the lowering of the viscosity, allows for the release of fluoride ions more easily than at an alkaline pH.

The profiles of fluoride release that were obtained at pH 4.5 showed very fast kinetics and a regularity was observed, indicating that, as the pH value increased, there was a lower concentration of fluoride ions in the tested medium. A similar effect was observed in an in vitro study evaluating fluoride release from commercially-available fluoride varnishes (Fluor Protector, Duraphat, and Clinpro White Varnish) [47]. It was found that the level of fluoride release was the greatest during the first phase of the experiment.

It may be that the fluoride ions released from NaF were incorporated into the structure of the fluorapatite. Earlier studies [48] indicate that, at an acidic pH value (pH 4 and 5), the hydroxyl groups present in nFAP are replaced by F ions, which is sufficient to reduce solubility and lead to a phase with greater crystallinity. This promotes a slower and more sustained release of fluoride and may contribute to its prolonged mineralizing effects on the enamel. Due to the low solubility of nFAP (pKso = 60.15 at 37 °C [49]), no fluoride ions were observed in the artificial saliva in the case of the G-nFAP gel, which did not contain NaF.

In conclusion, a low pH significantly favors the release of fluoride ions from hydrogels; this effect is related to the characteristics of the gel matrix, as its stability is pH dependent. The reduction in the pH affects the destabilization of the Carbopol gel, which results in a loose polymer network structure and leads to easier fluoride release. Similar results can be observed in previous studies that have researched the effect of pH on the fluoride release of various restorative materials. A.A. Taqa [50] and R.N. Bahadure [51] found that the amount of fluoride released from three restorative materials was significantly higher in acidic solutions than in neutral solutions, confirming the positive correlation between the amount of fluoride released and a low pH value that we observed in this study.

Another factor that influences the level of released fluoride is the presence of fluorapatite. Independently of the pH of the artificial saliva, its addition to the hydrogel significantly lowered the rate of fluoride release. At a pH of 4.5, representing the conditions during increased acidity and the demineralization of hard dental tissues, the level of released fluoride in the G-F gel was around 180 µg/cm^2^, whereas, in the G-F-nFAP gel, the amount of fluoride released only reached up to 95 µg/cm^2^ after the first 6 h of the experiment. This outcome can be attributed to the altered physical properties of the hydrogel, as well as possible chemical interactions between fluoride ions and nFAP. The rheological tests that were performed on the gels show that adding nFAP to the hydrogel increased its structural stability and viscosity, thereby decreasing the diffusion parameters of the gel. This observation goes back to the Einstein–Stokes equation [52], which shows the negative proportional relationship of viscosity and diffusion. If a given particle diffuses through a very dense or viscous medium, the process is hampered and takes significantly longer compared to the diffusion through a medium with a lower viscosity. Therefore, fluoride ions are released less easily and in lower amounts from the more viscous hydrogel G-F-nFAP.

The presence of nFAP may also have resulted in chemical interactions with the NaF particles of the gel. A previous study showed that the release of fluoride ions from sodium fluoride altered when other ions were dissolved in the same solution, emphasizing the high interactivity of NaF [53]. The incorporation of the fluoride ions into the structure of nFAP may have resulted in a lowered level of fluoride release from the material; however, neither the degree of incorporation nor the ion exchange reaction were further assessed in this study.

Examining fluoride release into artificial saliva from Duraphat varnish, Milburn et al. [54] showed no detectable fluoride ion emission at three weeks; meanwhile, in our study, the hydrogels still released fluoride after four weeks, particularly at a low pH (Figure 6).

This observation could confirm the benefits of choosing a hydrogel as a base material for topical fluoride treatments of dental hypersensitivity.

## 3. Conclusions

The presented results apply to hydrogels containing desensitizing agents (nanohydroxyapatite and sodium fluoride) that are used in the management of dentin hypersensitivity. The varying viscosities of the formulations influenced the process of fluoride release. The gel marked as G-F showed the fastest release of fluoride ions. It should be mentioned that the pH value of the medium is an important parameter in differentiating the obtained results of fluoride ion release. The release tendency is clear, i.e., the amount of fluoride ions released increases with the decrease in the pH value. This is related to the characteristics of the gel matrix, as the stability is pH dependent. Reducing the pH affects the destabilization of the Carbopol gel, which leads to a loose polymer network structure and easier release.

The amounts of free water and water bound to the hydrogel determine the solubility of the substance in the hydrogel and its release of drugs.

## 4. Materials and Methods

### 4.1. Materials

Calcium nitrate tetrahydrate (Ca(NO_3_)_2_·4H_2_O, 99%) was obtained from Acros Organics (Geel, Belgium). Ammonium fluoride (NH_4_F, 98%) was purchased from Alfa Aesar (Haverhill, MA, USA). Diammonium phosphate ((NH_4_)_2_HPO_4_, 98%) was obtained from Avantor Performance Materials (Gliwice, Poland). Sodium fluoride (NaF, ≥99%), glycerol (C_3_H_8_O_3_, ≥99.5%), and sodium hydroxide (NaOH, ≥97.0%) were purchased from Merck (St. Luis, MO, USA). Carbopol 974 was obtained from B. F. Goodrich Specialty Chemicals (Cleveland, OH, USA). All other reagents were of analytical grade. Deionised water was obtained using an Hydrolab Ultra UV (Hydrolab, Straszyn, Poland) ion-exchange system and it was used for the preparation of all solutions.

#### 4.1.1. Nanosized Fluorapatite Synthesis

Nanosized fluorapatite was synthesized using the co-precipitation method. The starting materials were calcium nitrate tetrahydrate (Ca(NO_3_)_2_·4H_2_O), ammonium fluoride (NH_4_F), diammonium phosphate ((NH_4_)_2_HPO_4_), and ammonia solution for pH adjustment.

Stoichiometric amounts of the starting materials were dissolved in deionized water. Then, the solutions were mixed, and synthesis was carried out on a magnetic stirring plate (Heidolph Instruments GmbH & CO. KG, Schwabach, Germany) at 100 °C for 1.0 h. The reaction was maintained at pH ~10, adjusted with aqueous ammonia. The precipitate obtained was washed and centrifuged until a neutral pH was reached, but not less than three times. Finally, the materials were dried for 24 h at 70 °C and were later heat-treated at 450 °C for 6 h to form crystallized nanoparticles.

#### 4.1.2. Procedure for the Preparation of Hydrogels

In the first step, the gel matrix was prepared on based Carbopol 974, after the polymer was dispersed in deionized water at 25 °C (using a defined amount of polymer and water, so that the final formulations contained 1.5% (*w/w*) of polymer (Table 3)). The modified preparation of the Carbopol gel was based on the procedure published by P. R. Varges [55].

After 5 h, the dispersion was homogenized using a Heidolph MR Hei-Standard (Heidolph Instruments GmbH&Co, Germany) magnetic stirrer and then degassed. The glycerol (10 g per 100 g final gel) was used to give stretchability [56] and, as a biodegradable material, is compatible with the principles of green chemistry [57]. For the stability [58], the gel matrix was stirred for 1 week. Bubble formation was minimized by carefully dispersing the polyacrylic polymer [59].

**Figure 7:**
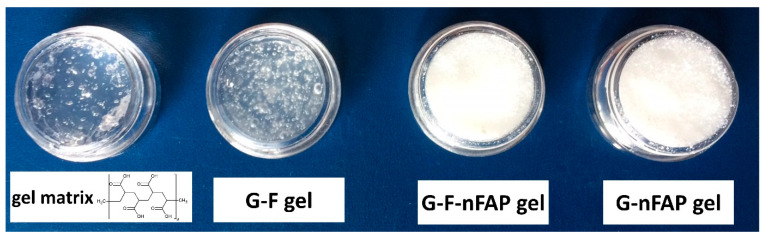
The hydrogel formulations based on a 1.5% *w/w* Carbopol matrix and the structure of the Carbopol polymer.

The Carbopol matrix was used to obtain the hydrogel formulations G-F, G-F-nFAP, and G-nFAP containing NaF and nFAP (Table 3, Figure 7). Then, the NaF solution was prepared by dissolving NaF (4 g) in distilled water. The dispersion of nFAP was obtained after 15 min sonification of 10 g nFAP in water. The aqueous dispersions of the Carbopol polymers were neutralized with 10% sodium hydroxide solution to pH 7.0 in order to achieve maximum viscosity (the kneading method). After measurement, the hydrogels were stabilized at 25 or 37 °C for 12 h. The pH values of the gels were measured using a combined glass electrode (pH & GLP22 + Crison ionometer, Spain) three times for each sample at a dilution of 5.0 g in 20 mL of deionized water.

### 4.2. Methods

#### 4.2.1. Physicochemical Analysis of the Fluorapatite Compound

The crystal phase purity of fluorapatite was analyzed using the X-ray diffraction (XRD) method. The obtained pattern was collected with an X’Pert PRO X-ray diffractometer (CuKα, 1.54060Å) (PANalytical, Malvern Panalytical Ltd., Malvern, UK). The diffractogram was analyzed and assigned to the standard pattern from the Inorganic Crystal Structure Database (ICSD). Analysis of the morphology and size of the fluorapatite powder was performed on the SEM (scanning electron microscope) FEI Nova NanoSEM 230 (Hillsboro, OR, USA), which was equipped with an energy-dispersive (EDS) spectrometer (EDAX Genesis XM4).

#### 4.2.2. Rheological Measurements

Rheological measurements were carried out using a ViscotesterTM iQ (Haake Instruments Inc.) with a temperature module (liquid controlled). In order to perform the flow curve tests and viscosity tests, a titanium plate–plate-type geometrical arrangement (20 mm) was used in the range of shear rate 1–300 s^−1^ with a 1mm gap between the plates. The measurement was started after the stabilization of samples for 30 min while controlling the temperature of the lower plate. The dependence of the viscosity on the temperature of the gels was measured in the range of 25–37.5 °C. Rheograms were recorded at 25 and 37 °C. The formulations were analyzed using the rheological models [59]. The experimental tests were programmed using Thermo Scientific™ HAAKE™ RheoWin™ Software version 4.30.

#### 4.2.3. FT-IR Spectroscopy Measurement

The spectra of the Carbopol gels were recorded on a Thermo Scientific Nicolet iS50 FT-IR spectrometer (Waltham, MA USA) (with an attenuated total reflection method, or ATR method) with a DTGS detector and a KBr beam splitter. The ATR FT-IR spectra were collected in the 400 and 4000 cm^−1^ regions, at room temperature, with a spectral resolution of 4 cm^−1^ and 32 scans.

#### 4.2.4. Swelling Test

The hydrogels were prepared according to the procedure described in Section 4.1.2 and, after 1 week, the gels were lyophilized (at −40 °C in a freeze-dryer, Alfa 1–2 LD plus, Christ, Germany); after this, swelling tests were performed using the filtration method [60] and the Fusayama–Meyer artificial saliva buffer (100 mL) at 3 different pH values (4.5, 6.6, and 8.0) and at a temperature of 37 °C. The dry samples (with the initial weight *W_d_* = 0.4–0.5 g) were removed at 10 min time intervals (within the first hour of the experiment) and at 1-h intervals (between 1 and 6 h of the experiment). The water was removed with a vacuum pump and the samples were weighed (*W_s_*). The same procedure was also carried out for the filter paper (*W_o_*) for filtration. The swelling index of the hydrogel samples was determined using the following equation (Equation (2)):

(2) $$Swelling\;index=\frac{W_{s}-W_{o}-W_{d}}{W_{d}}$$

where *W_s_*, *W_o_*, and *W_d_* are the weights of the swollen probes, the pre-saturated filter paper, and dry samples after lyophilization, respectively. The swelling test was performed in triplicate.

#### 4.2.5. Thermogravimetric Analysis

TGA thermograms of the hydrogel samples were examined using a TG 209F1 Libra instrument (Netzsch, Germany). The gels were analyzed from 25 to 1000 °C with a 10 °C/min heating rate and a nitrogen atmosphere (at flow rate of 15 mL/min). The samples’ mass range was 3.5–5.0 mg.

#### 4.2.6. In Vitro Study of Fluoride Release from Hydrogels

Fluoride release studies from 3 hydrogels (G-F gel, G-F-nFAP gel, and G-nFAP gel, containing 20,000 ppm fluoride ions; a mixture of fluoride ions 20,000 ppm and 10% (*w/w*) fluorapatite; and 10% (*w/w*) fluorapatite, respectively) were performed in 3 solutions imitating the composition of human saliva at pH values of 4.5, 6.6, and 8.0. 

The Fusayama–Meyer artificial saliva [61] was composed of a variety of chemical compounds with buffering properties, such as NaCl (0.4 g/L), KCl (0.4 g/L), CaCl_2_·2H_2_O (0.795 g/L), urea (1 g/L), Na_2_S·9H_2_O (0.005 g/L), and NaH_2_PO_4_·2H_2_O (0.78 g/L). The values of the pH of the saliva were determined using a combined glass electrode (by pH&Ion-meter GLP22 + Crison) and adjusted to the expected pH with 2 M/L HCl or 2 M/L NaOH solutions. Before the start of the experiment, the gels (5 g) were put into cells with a dialysis membrane (SERVAPOR^®^) with a pore diameter of approx. 25 Å, to allow for contact between the gel components and the medium. The cells with gels were immersed in polyethylene bottles (with screw caps) with the reference medium (100 mL). Samples were incubated at 37 °C with stirring at 100 rpm. Aliquots of 1 mL were withdrawn by a pipette and replaced immediately with 1 mL of the fresh medium. The samples were evaporated, and then, after dissolving in 1 mL 2M/L HCl to dissolve calcium compounds (fluoride, phosphates, and hydroxide), the probes were adjusted to pH 5.5 with a TISAB buffer (a total ionic strength adjustment buffer). A time-dependent release study was carried out for 0–5 h (with 1-hour intervals), then after 24 and 48 h, and finally after 1–5 weeks (with 1-week intervals). The determination of the fluoride concentration was measured by direct potentiometry using a Multifunction computer meter MCM CX-731 (Elmetron, Poland) with a fluoride-ion-selective electrode (09–37 type, Marat) and a reference electrode of Ag/AgCl (RAE 111 type, Marat). The measurement was based on the standard curve in the range 1 × 10^−6^–1 × 10^−2^ M/L. All experiments were performed in triplicate and the results are expressed as ppm. The fluoride ion release % was calculated using the following formula (Equation (3)):

(3) $$Fluoride\;ions\;release\;\left(\%\right)=\frac{mass\;of\;fluoride\;ions\;released\;at\;time}{total\;mass\;of\;fluoride\;ions\;at\;the\;hydrogel}\times 100$$

The amount of fluoride ions in the saliva was calculated relative to the active surface of the membrane (1.77 cm^2^).

### 4.3. Statistical Analysis

The mean values of concentrations of fluoride ions were calculated for 6 formulations of hydrogels in triplicate. The results are presented as mean ± standard deviation values. All measurements were made in triplicate.

## Figures and Tables

**Table 1:** Atomic parameters of the Ca_10_(PO_4_)_6_F_2_.

Sample	Ca_10_(PO_4_)_6_F_2_; Z = 2					
**Space group**	Hexagonal P6_3_/m (No. 176)					
**Calculated cell parameters**	a = 9.382(0) Å c = 6.892(1) Å V = 525.37(8) Å^3^					
R_w_ R_wnb_ R_all_ R_nb_ σ	2.87% 2.35% 2.22% 2.08% 1.32%					
**Selected shortest contacts**						
Ca–Ca Ca–O Ca–P P–O Ca–O–Ca	4.0049(33) Å 2.3562(64) Å 3.2043(73) Å 1.5294(86) Å 111.823(276)°					
**Atom**	**Wyckoff positions**	**x**	**y**	**z**	**B_iso_**	**Occ. (<1)**
Ca_1_	4 f	0.3333	0.6665	0.00064	0.690871	0.989
Ca_2_	6 h	0.2408	0.9822	0.2498	0.412068	0.986
P_1_	6 h	0.3981	0.3682	0.25	0.709863	1.007
O_1_	6 h	0.3224	0.4796	0.2499	0.318567	987
O_2_	6 h	0.5904	0.4687	0.2498	0.218500	987
O_3_	12 i	0.3339	0.2510	0.0644	0.254603	988
F_1_	2 a	0	0	0.2499	0.030193	0.906

**Table 2:** The yield points of the G-F, G-F-nFAP, and G-nFAP hydrogels at 25 and 37 °C. The range of the shear rate was 1–300 s^−1^.

Hydrogel	Yield Point (Pa) at 25 °C	Yield Point (Pa) at 37 °C
G-F	142.55 ± 10.09	139.21 ± 9.05
G-F-nFAP	178.01 ± 9.13	172.15 ± 10.03
G-nFAP	182.34 ± 12.20	178.48 ± 8.15

**Table 3:** Compositions of the prepared hydrogels in (g) per 100 g gel.

Hydrogels	Carbopol 974 (g)	86% Glycerol (g)	Sodium Fluoride (g)	nFAP (g)	10% Sodium Hydroxide (g) *	Water (g)
G-F	1.5	10	4	-	6.4	78.1
G-F-nFAP	1.5	10	4	10	2.8	71.7
G-nFAP	1.5	10	−-	10	6.2	72.3

* to pH 7.0.

## Data Availability

Not applicable.

## Supplementary Materials

The following supporting information can be downloaded at: https://www.mdpi.com/article/10.3390/gels9040271/s1, Figure S1: Rheograms of gels G-F, G-F-nFAP, and G-nFAP at 25 (A) and 37 °C (B), 1 week after preparation. Shear stress as a function of shear rate; Figure S2. TGA thermograms of the G-F gel, G-F-nFAP gel, G-nFAP gel, and nFAP.
