# Supplementary material for: Nanofluorapatite Hydrogels in the Treatment of Dentin Hypersensitivity: A Study of Physiochemical Properties and Fluoride Release
Source: Gels. 2023 Mar 25;9(4):271. doi: 10.3390/gels9040271 (PMC10137577; doi:10.3390/gels9040271)
Supplement: Supplementary file 1 [file gels-09-00271-s001.zip › gels-2235379-supplementary.pdf]

gels

MDPI

Article

Nanofluorapatite Hydrogels in the Treatment of Dentin Hypersensitivity: A Study of Physiochemical Properties and Fluoride Release

Katarzyna Wiglusz [1,*], Maciej Dobrzynski [2], Martina Gutbier [2] and Rafal J. Wiglusz [3,*]

Supplementary Materials

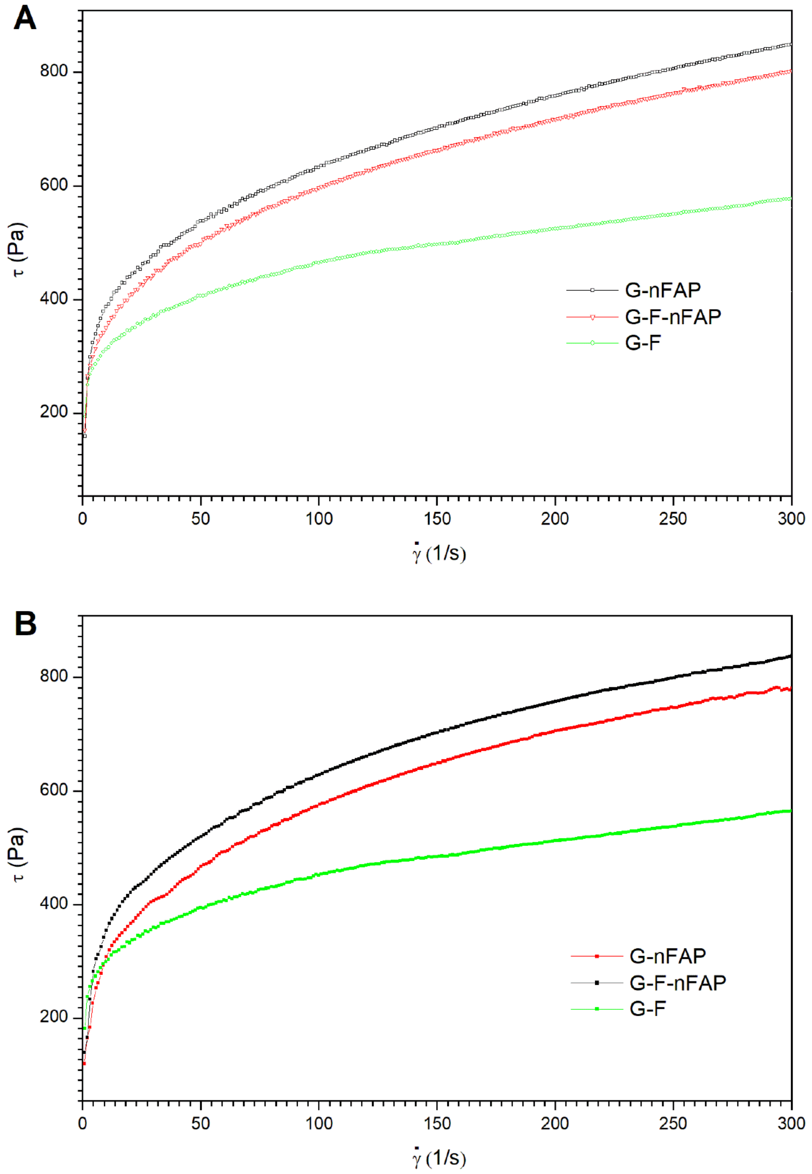

Figure S1. Rheograms of gels G-F, G-F-nFAP, G-nFAP at 25 (**A**) and 37°C (**B**), 1 week after preparation. The shear stress as a function of shear rate.

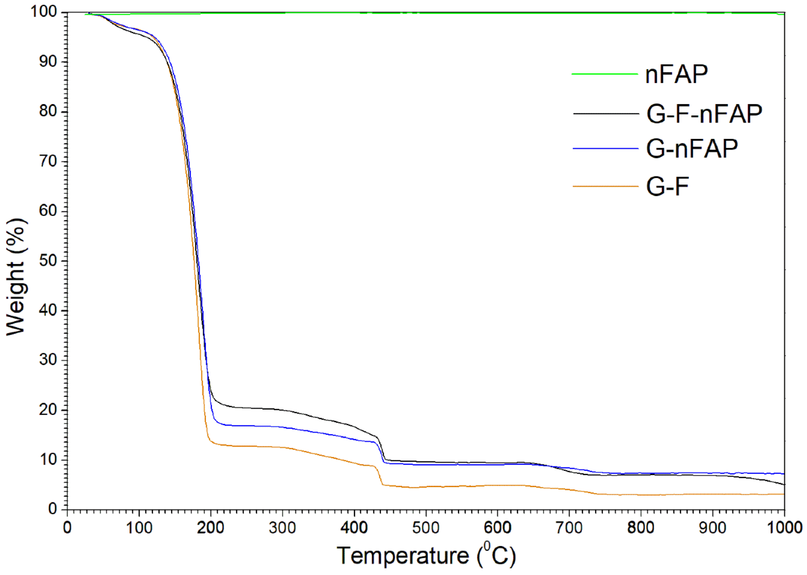

Figure S2. TGA thermograms of the G-F gel, G-F-nFAP gel, G-nFAP gel, G nFAP, and FAP.
